# Advances in infection control

**DOI:** 10.1590/S1679-45082016MD3433

**Published:** 2016

**Authors:** Alexandre Rodrigues Marra

**Affiliations:** 1 Hospital Israelita Albert Einstein, São Paulo, SP, Brazil.

**Keywords:** Cross infection/prevention & control, Anti-infective agents, local, Hand hygiene, Health knowledge, attitudes, practice

## Abstract

Several initiatives took place in recent years in relation to nosocomial infection control in order to increase patient safety. Some of these initiatives will be commented in this brief review.

## INTRODUCTION

Nosocomial infections such as ventilator-associated pneumonia and central venous catheter-related bloodstream infection are important causes of morbidity and mortality. Implementation of prevention measures for these infections have showed zero infection rate in a number of intensive care units,^1^ as well as at different hospital units.^2^

Changes in health professional behavior,^3^ who actively participate in reducing infection rates, have reduced morbidity, mortality, related costs and have also shown more safety for patients.

In addition, the increase of participation, the implementation of new technologies, such as antibacterial or antiseptic-impregnated invasive devices, the use of ultrasound resource for central venous catheter insertion, and chlorhexidine-impregnated dressing are welcoming resources for better practice not only for physicians but also to all multidisciplinary health team responsible for patients’ care.^4^

Despite all innovations nosocomial infection, hand hygiene is still the most important procedure for preventing infections in hospitals.^5^

Health professionals often complain about the difficult for hand hygiene. The major complaints are related with handwashing problems, such as dry skin and injuries caused by soap or detergent, and other reasons. Professionals report that handwashing procedure is time-consuming and cause interruption in routine patient care tasks.^5^ Many studies have shown that frequent and repetitive handwashing (with soap and water, but in this case with chlorhexidine), in a number of American and European hospitals present compliance rates below 50%.^6,7^

Currently, strategies to increase compliance with handwashing are focused on reduction of time needed for this task. International guidelines for infection prevention related with intravascular catheters emphasize hand hygiene.^8^

Daily there are a number of opportunities for non-compliance to hand hygiene in hospital units particularly because of high-complexity patients. However, because patients are hospitalized in private rooms, great difficulties exist to measure hand hygiene compliance.^3,5^

An alternative for conventional hand antisepsis is the use of alcohol-based hand rubs. Hand hygiene compliance rate increased when chlorhexidine was replaced by alcohol-based hand rubs.^5,6^ Several studies reported that hand hygiene compliance is poor among health professionals.^3-7^

Recent studies have showed that include technologies such as electronic counters and video camera monitoring in order to give real-time feedback for professionals regarding hand hygiene have increased compliance.^9^ In our hospital, we use a radio frequency identification, which does not require wireless internet, named ZigBee (i-Healthsys, São Carlos, Brazil). This system enables to monitor handwashing, without the need of human observation. The system interacts with health professional by a light flashing. The use of this new technology increased hand hygiene compliance at our institution.^10^ In addition, this technology may bring benefit to our patients and we believe this procedure should be applied in other hospitals.

A number of side effects may occur in hospitals, among them are infectious events that in the past were considered expected and preventable. Currently, these events are not accepted and many of them, such ventilator-associated pneumonia and central venous catheter-related bloodstream infection, are no longer covered by the American health system (Medcare and Medcaid).^1,2,4^ Unfortunately in Brazil this is not a reality yet, but, perhaps, in the near future it will be.
