# Supplementary material for: Advances in infection control
Source: Einstein (Sao Paulo). 2016 Jan-Mar;14(1):108–9. doi: 10.1590/S1679-45082016MD3433 (PMC4872927; doi:10.1590/S1679-45082016MD3433)
Supplement: Translation

# Translation: Avanços no controle das infecções

**Authors:** Alexandre Rodrigues Marra

**Affiliations:** 1 Hospital Israelita Albert Einstein, São Paulo, SP, Brasil.

**Keywords:** Infecção hospitalar/prevenção & controle, Anti-infecciosos locais, Higiene das mãos, Conhecimentos, atitudes e prática em saúde

## Abstract

Várias iniciativas aconteceram nos últimos anos em relação ao controle das infecções no ambiente hospitalar para aumentar a segurança do paciente. Algumas dessas iniciativas são comentadas nesta breve revisão.

## INTRODUÇÃO

Infecções adquiridas no ambiente hospitalar, como a pneumonia associada à ventilação mecânica e a infecção da corrente sanguínea associada ao cateter venoso central, são importantes causas de morbidade e mortalidade. A implementação de pacotes de prevenção para essas infecções vem apresentando taxas zero de infecção em diversas unidades de terapia intensiva,^1^ bem como em diferentes unidades hospitalares.^2^

A mudança de comportamento dos profissionais da saúde,^3^ que participam de forma ativa da diminuição das taxas das infecções, tem proporcionado diminuição de morbidade, mortalidade e custos, trazendo maior segurança para os pacientes.

Associada a essa maior participação, a incorporação de novas tecnologias, como dispositivos invasivos impregnados com antissépticos ou antimicrobianos, utilização do recurso de ultrassom para passagem do cateter venoso central, curativos impregnados com clorexidina no cuidado ao cateter venoso central e banho com clorexidina, é bem-vinda para as melhores práticas, não somente para os médicos, mas para toda a equipe multiprofissional que presta assistência ao paciente.^4^

Apesar de todas essas inovações no campo da infecção hospitalar, a higiene das mãos ainda é o procedimento mais importante para a prevenção das infecções hospitalares.^5^

Profissionais da saúde frequentemente se queixam das dificuldades para a higiene das mãos; as principais queixas estão relacionadas a problemas com o ato da lavagem, como pele seca e lesão nas mãos pelo sabão ou detergente, e também por outros motivos, como o gasto de tempo e a interrupção de procedimentos rotineiros de cuidados ao paciente.^5^ Muitos estudos têm demonstrado que a lavagem de mãos (com água e sabão, aqui, no caso, a clorexidina), de forma regular e repetidamente em muitos hospitais americanos e europeus apresenta taxas menores do que 50%.^6,7^

Atualmente, as estratégias para aumento da adesão à lavagem das mãos estão direcionadas à redução do tempo necessário para esse procedimento. Diretrizes internacionais de prevenção de infecção relacionada a cateteres intravasculares destacam a higiene das mãos.^8^

Diariamente acontecem diversas oportunidades para a não adesão à higienização das mãos nas unidades hospitalares, em virtude da alta complexidade dos pacientes. Porém, pelo fato de os pacientes ficarem hospitalizados em quartos fechados, existe uma grande dificuldade para se quantificar a adesão à essa prática de higienização das mãos.^3,5^

Uma opção à antissepsia convencional é a utilização de compostos de gel alcoólico. Observou-se que ocorreu um aumento na taxa de adesão à higiene das mãos quando a clorexidina foi substituída por gel alcoólico.^5,6^ Vários estudos documentaram que as taxas de higienização das mãos pelos profissionais de saúde são insatisfatórias.^3-7^

Recentes estudos demonstram que a incorporação de tecnologias, como contadores eletrônicos e monitorização por vídeo, no intuito de darem *feedback* ao profissional em relação à higiene das mãos, aumentou sua aderência.^9^ Em nosso hospital, utilizamos o recurso de monitorização por radiofrequência (RFID - *RadioFrequency Identification*, que utiliza uma radiofrequência sem necessidade de *wi-fi*, chamada *zigbee –* [i-Healthsys, São Carlos, Brasil]). Por esse sistema, é possível monitorar a higiene das mãos sem a necessidade de um observador humano, e com interação com o profissional da saúde por meio de um aviso luminoso. Incorporando essas novas técnicas, conseguimos aumentar a aderência para a higiene das mãos.^10^ Assim, deve haver benefício para os pacientes e para a reprodutibilidade em outros hospitais.

Vários eventos adversos podem ocorrer nos hospitais, entre eles eventos infecciosos, que até anos atrás eram considerados esperados e previsíveis. Hoje em dia, são considerados inadmissíveis e muitos deles, como a pneumonia associada à ventilação mecânica e a infecção da corrente sanguínea associada ao cateter venoso central, não são mais reembolsáveis pelo sistema de saúde americano (Medcare e Medcaid).^1,2,4^ Isso ainda não é uma realidade em nosso país. Poderá ser em um futuro próximo.
